# (Diphenyl­phosphor­yl)(2-nitro­phen­yl)methanol

**DOI:** 10.1107/S160053681003196X

**Published:** 2010-08-18

**Authors:** Xiao-Ling Yuan, Wan-Yun Liu, Ping Huo, Guang-Quan Mei

**Affiliations:** a Key Laboratory of Jiangxi University for Applied Chemistry and Chemical Biology, Yichun 336000, People’s Republic of China

## Abstract

In the title compound, C_19_H_16_NO_4_P, the dihedral angle between the mean planes of the phenyl rings bonded to the P atom is 75.4 (1)°. In the crystal, mol­ecules are linked into chains running along the *a* axis by inter­molecular O—H⋯O hydrogen bonds. Mol­ecules are further connected into a three-dimensional array by weak C—H⋯O hydrogen bonds.

## Related literature

For applications of the analogous compound (diphenyl­phosphino­yl)phenyl­methanol in the rhodium-catalysed hydro­formyl­ation of alkenes, see: Clark *et al.* (2002 ▶). For related structures, see: Liu *et al.* (2007 ▶); Liu & Huo (2008 ▶).
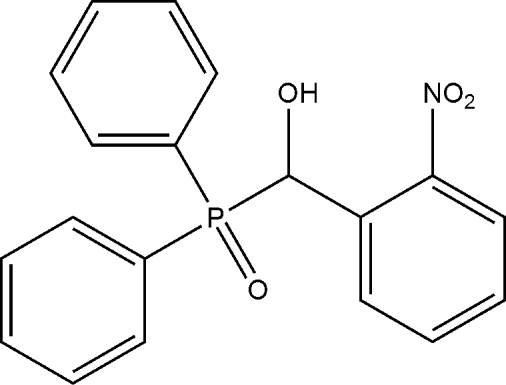


## Experimental

### 

#### Crystal data

C_19_H_16_NO_4_P
*M* *_r_* = 353.30
Orthorhombic, 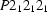

*a* = 5.9179 (12) Å
*b* = 13.917 (3) Å
*c* = 20.405 (4) Å
*V* = 1680.6 (6) Å^3^
*Z* = 4
Mo *K*α radiation
μ = 0.19 mm^−1^
*T* = 293 K
0.35 × 0.22 × 0.13 mm

#### Data collection

Bruker SMART APEX area-detector diffractometer
Absorption correction: multi-scan (*SADABS*; Bruker, 2001 ▶) *T*_min_ = 0.533, *T*_max_ = 1.000
14520 measured reflections
3311 independent reflections
3065 reflections with *I* > 2σ(*I*)
*R*_int_ = 0.028

#### Refinement

*R*[*F* ^2^ > 2σ(*F* ^2^)] = 0.029
*wR*(*F* ^2^) = 0.078
*S* = 1.05
3311 reflections
226 parameters
H-atom parameters constrained
Δρ_max_ = 0.18 e Å^−3^
Δρ_min_ = −0.20 e Å^−3^
Absolute structure: Flack (1983 ▶), 1377 Friedel pairs
Flack parameter: 0.19 (7)

### 

Data collection: *SMART* (Bruker, 2001 ▶); cell refinement: *SAINT* (Bruker, 2001 ▶); data reduction: *SAINT*; program(s) used to solve structure: *SHELXS97* (Sheldrick, 2008 ▶); program(s) used to refine structure: *SHELXL97* (Sheldrick, 2008 ▶); molecular graphics: *ORTEP-3 for Windows* (Farrugia, 1997 ▶); software used to prepare material for publication: *SHELXL97*.

## Supplementary Material

Crystal structure: contains datablocks I, global. DOI: 10.1107/S160053681003196X/pb2037sup1.cif

Structure factors: contains datablocks I. DOI: 10.1107/S160053681003196X/pb2037Isup2.hkl

Additional supplementary materials:  crystallographic information; 3D view; checkCIF report

## Figures and Tables

**Table 1:** Hydrogen-bond geometry (Å, °)

*D*—H⋯*A*	*D*—H	H⋯*A*	*D*⋯*A*	*D*—H⋯*A*
O2—H2*A*⋯O1^i^	0.82	1.86	2.6483 (16)	161
C4—H4*A*⋯O3^ii^	0.93	2.52	3.207 (2)	131
C19—H19*A*⋯O2^iii^	0.93	2.50	3.404 (2)	164

Symmetry codes: (i) 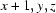; (ii) 
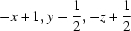
; (iii) 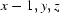.

**Fig. 1.**
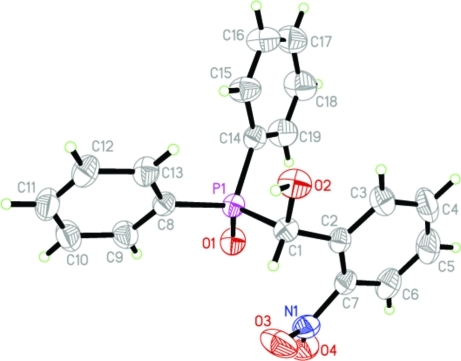
The molecular structure of (I), showing 50% probability displacement ellipsoids (arbitrary spheres for H atoms).

**Fig. 2.**
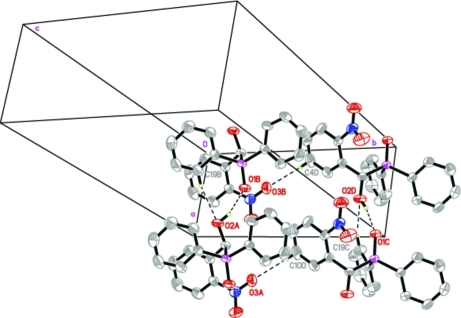
Part of the packing of the title compound. Intermolecular hydrogen bonds are represented by dashed lines. H atoms not involved in hydrogen bonding have been omitted.

## supplementary crystallographic information

### Comment

The title compound, (I), is an analog of (diphenylphosphinoyl)phenylmethanol, which was employed as a ligand in the rhodium-catalyzed hydroformylation of alkenes, with good conversions and regioselectivities (Clark *et al.*, 2002).

The molecular structure of (I) is shown in Fig. 1. Bond lengths and angles in (I) are in agreement with those reported for similar compounds (Liu *et al.*, 2007; Liu *et al.*, 2008). The dihedral angle between the mean-planes of the phenyl rings (C8—C13) and (C14—C19) bonded to P-atoms is 53.0 (1)°. The strong O—H···O and weak C—H···O intermolecular hydrogen bonds play a significant role in stabilizing the crystal structure; see Table 1 for geometric parameters and symmetry operations. A strong O—H···O hydrogen bond involving the hydroxyl group link the molecules into a chain running along the *a* axis. Molecules are further connected into a three-dimensional array by non-classical and rather weak C—H···O intermolecular hydrogen-bonding interactions.

### Experimental

To a solution of 2-nitrobenzaldehyde (0.30 g, 2.0 mmol) and diphenylphosphine oxide (0.40 g, 2.0 mmol) in tetrahydrofuran (10 ml) at 273 K was added dropwise triethylamine (0.03 ml, 2 mmol). The cooling bath was removed and the mixture warmed to ambient temperature for 2 h. The solvent was concentrated under vacuum and the crude product was purified by recrystallization in methanol to give the title compound as a white solid in 82% yield. Single crystals of (I) were obtained by slow evaporation of a methanol solution.

### Refinement

All H atoms were placed in geometrically idealized positions and constrained to ride on their parent atoms, with C—H = 0.93 Å (aromatic), 0.98 Å (methine), O—H = 0.82 Å, and *U*_iso_(H) = 1.2*U*_eq_(*c*) and 1.5*U*_eq_(O).

### Crystal data

**Table:**

C_19_H_16_NO_4_P	*F*(000) = 736
*M**_r_* = 353.30	*D*_x_ = 1.396 Mg m^−^^3^
Orthorhombic, *P*2_1_2_1_2_1_	Mo *K*α radiation, λ = 0.71073 Å
Hall symbol: P 2ac 2ab	Cell parameters from 2260 reflections
*a* = 5.9179 (12) Å	θ = 3.3–27.5°
*b* = 13.917 (3) Å	µ = 0.19 mm^−^^1^
*c* = 20.405 (4) Å	*T* = 293 K
*V* = 1680.6 (6) Å^3^	Plate, colorless
*Z* = 4	0.35 × 0.22 × 0.13 mm

### Data collection

**Table:**

Bruker APEX area-detector diffractometer	3311 independent reflections
Radiation source: fine-focus sealed tube	3065 reflections with *I* > 2σ(*I*)
graphite	*R*_int_ = 0.028
φ and ω scans	θ_max_ = 26.0°, θ_min_ = 3.1°
Absorption correction: multi-scan (*SADABS*; Bruker, 2001)	*h* = −7→7
*T*_min_ = 0.533, *T*_max_ = 1.000	*k* = −16→17
14520 measured reflections	*l* = −25→25

### Refinement

**Table:**

Refinement on *F*^2^	Secondary atom site location: difference Fourier map
Least-squares matrix: full	Hydrogen site location: inferred from neighbouring sites
*R*[*F*^2^ > 2σ(*F*^2^)] = 0.029	H-atom parameters constrained
*wR*(*F*^2^) = 0.078	*w* = 1/[σ^2^(*F*_o_^2^) + (0.0524*P*)^2^ + 0.0539*P*] where *P* = (*F*_o_^2^ + 2*F*_c_^2^)/3
*S* = 1.05	(Δ/σ)_max_ < 0.001
3311 reflections	Δρ_max_ = 0.18 e Å^−^^3^
226 parameters	Δρ_min_ = −0.20 e Å^−^^3^
0 restraints	Absolute structure: Flack (1983), 1373 Friedel pairs
Primary atom site location: structure-invariant direct methods	Flack parameter: please supply

### Special details

**Table:**

Geometry. All e.s.d.'s (except the e.s.d. in the dihedral angle between two l.s. planes) are estimated using the full covariance matrix. The cell e.s.d.'s are taken into account individually in the estimation of e.s.d.'s in distances, angles and torsion angles; correlations between e.s.d.'s in cell parameters are only used when they are defined by crystal symmetry. An approximate (isotropic) treatment of cell e.s.d.'s is used for estimating e.s.d.'s involving l.s. planes.
Refinement. Refinement of *F*^2^ against ALL reflections. The weighted *R*-factor *wR* and goodness of fit *S* are based on *F*^2^, conventional *R*-factors *R* are based on *F*, with *F* set to zero for negative *F*^2^. The threshold expression of *F*^2^ > σ(*F*^2^) is used only for calculating *R*-factors(gt) *etc*. and is not relevant to the choice of reflections for refinement. *R*-factors based on *F*^2^ are statistically about twice as large as those based on *F*, and *R*- factors based on ALL data will be even larger.

### Fractional atomic coordinates and isotropic or equivalent isotropic displacement parameters (Å^2^)

**Table:**

	*x*	*y*	*z*	*U*_iso_*/*U*_eq_	
P1	0.36544 (6)	0.05040 (3)	0.12304 (2)	0.02988 (11)	
O1	0.13285 (17)	0.06076 (8)	0.15090 (6)	0.0399 (3)	
O2	0.75302 (16)	−0.03926 (10)	0.14345 (6)	0.0448 (3)	
H2A	0.8541	−0.0008	0.1517	0.067*	
O3	0.3080 (3)	0.04117 (11)	0.29316 (8)	0.0683 (4)	
O4	−0.0305 (2)	−0.01286 (11)	0.29317 (8)	0.0624 (4)	
C1	0.5574 (2)	−0.01474 (11)	0.17954 (8)	0.0323 (3)	
H1A	0.5967	0.0257	0.2172	0.039*	
C2	0.4346 (3)	−0.10461 (11)	0.20185 (8)	0.0328 (3)	
C3	0.4930 (3)	−0.19195 (13)	0.17376 (10)	0.0461 (4)	
H3A	0.6165	−0.1948	0.1456	0.055*	
C4	0.3715 (4)	−0.27538 (13)	0.18661 (12)	0.0604 (6)	
H4A	0.4172	−0.3332	0.1680	0.072*	
C5	0.1858 (4)	−0.27310 (14)	0.22626 (12)	0.0622 (6)	
H5A	0.1040	−0.3290	0.2341	0.075*	
C6	0.1201 (4)	−0.18790 (13)	0.25448 (10)	0.0524 (5)	
H6A	−0.0076	−0.1856	0.2810	0.063*	
C7	0.2453 (3)	−0.10560 (12)	0.24313 (8)	0.0360 (4)	
C8	0.4845 (3)	0.16772 (11)	0.10671 (8)	0.0320 (3)	
C9	0.3434 (3)	0.24491 (12)	0.12017 (10)	0.0455 (4)	
H9A	0.2010	0.2337	0.1379	0.055*	
C10	0.4114 (4)	0.33817 (13)	0.10765 (11)	0.0552 (5)	
H10A	0.3158	0.3892	0.1177	0.066*	
C11	0.6187 (4)	0.35558 (13)	0.08061 (10)	0.0512 (5)	
H11A	0.6629	0.4183	0.0713	0.061*	
C12	0.7628 (3)	0.27996 (13)	0.06705 (10)	0.0472 (5)	
H12A	0.9037	0.2921	0.0487	0.057*	
C13	0.6988 (3)	0.18611 (12)	0.08065 (9)	0.0408 (4)	
H13A	0.7979	0.1357	0.0725	0.049*	
C14	0.3674 (3)	−0.02135 (11)	0.04912 (8)	0.0349 (3)	
C15	0.5420 (3)	−0.01949 (15)	0.00353 (10)	0.0496 (5)	
H15A	0.6643	0.0214	0.0100	0.060*	
C16	0.5353 (4)	−0.07759 (16)	−0.05106 (11)	0.0575 (5)	
H16A	0.6521	−0.0753	−0.0815	0.069*	
C17	0.3564 (4)	−0.13884 (15)	−0.06060 (11)	0.0591 (5)	
H17A	0.3526	−0.1784	−0.0973	0.071*	
C18	0.1833 (4)	−0.14165 (17)	−0.01602 (12)	0.0643 (6)	
H18A	0.0624	−0.1833	−0.0225	0.077*	
C19	0.1878 (3)	−0.08300 (14)	0.03839 (11)	0.0515 (5)	
H19A	0.0689	−0.0849	0.0682	0.062*	
N1	0.1687 (3)	−0.01963 (11)	0.27860 (7)	0.0446 (4)	

### Atomic displacement parameters (Å^2^)

**Table:**

	*U*^11^	*U*^22^	*U*^33^	*U*^12^	*U*^13^	*U*^23^
P1	0.02187 (17)	0.03298 (19)	0.0348 (2)	−0.00254 (14)	0.00264 (15)	0.00331 (17)
O1	0.0241 (5)	0.0443 (6)	0.0512 (7)	−0.0008 (5)	0.0076 (5)	0.0058 (5)
O2	0.0226 (5)	0.0599 (8)	0.0520 (8)	−0.0028 (5)	0.0049 (5)	−0.0046 (6)
O3	0.0721 (9)	0.0642 (9)	0.0686 (10)	−0.0235 (8)	0.0197 (8)	−0.0319 (9)
O4	0.0511 (8)	0.0733 (9)	0.0628 (10)	0.0023 (7)	0.0212 (7)	−0.0041 (8)
C1	0.0243 (7)	0.0376 (8)	0.0349 (8)	−0.0013 (6)	0.0013 (6)	0.0010 (7)
C2	0.0309 (7)	0.0336 (8)	0.0338 (8)	0.0009 (6)	−0.0053 (6)	0.0038 (7)
C3	0.0444 (9)	0.0395 (9)	0.0545 (11)	0.0069 (8)	−0.0028 (9)	−0.0016 (8)
C4	0.0753 (14)	0.0295 (8)	0.0763 (15)	0.0073 (10)	−0.0113 (13)	−0.0023 (9)
C5	0.0727 (14)	0.0372 (10)	0.0766 (16)	−0.0154 (10)	−0.0069 (12)	0.0133 (10)
C6	0.0526 (10)	0.0496 (10)	0.0549 (12)	−0.0129 (9)	0.0043 (10)	0.0149 (9)
C7	0.0380 (8)	0.0343 (8)	0.0355 (9)	−0.0040 (7)	−0.0001 (7)	0.0050 (7)
C8	0.0307 (7)	0.0333 (8)	0.0320 (8)	−0.0040 (6)	0.0008 (6)	0.0027 (6)
C9	0.0440 (8)	0.0400 (8)	0.0525 (11)	0.0002 (7)	0.0113 (9)	−0.0016 (8)
C10	0.0632 (12)	0.0354 (9)	0.0670 (14)	0.0054 (8)	0.0124 (10)	−0.0033 (9)
C11	0.0618 (11)	0.0381 (9)	0.0537 (11)	−0.0095 (9)	0.0003 (10)	0.0091 (8)
C12	0.0411 (9)	0.0485 (10)	0.0520 (12)	−0.0127 (8)	0.0048 (8)	0.0100 (9)
C13	0.0319 (8)	0.0401 (9)	0.0504 (11)	−0.0016 (7)	0.0052 (7)	0.0049 (8)
C14	0.0337 (7)	0.0370 (8)	0.0340 (8)	−0.0019 (7)	−0.0047 (7)	0.0027 (7)
C15	0.0442 (9)	0.0607 (11)	0.0438 (10)	−0.0113 (9)	0.0031 (8)	−0.0067 (9)
C16	0.0590 (12)	0.0719 (14)	0.0416 (11)	−0.0029 (10)	0.0068 (9)	−0.0070 (10)
C17	0.0732 (13)	0.0576 (11)	0.0465 (11)	−0.0026 (11)	−0.0087 (11)	−0.0119 (9)
C18	0.0615 (13)	0.0644 (13)	0.0670 (15)	−0.0209 (11)	−0.0051 (11)	−0.0163 (11)
C19	0.0440 (10)	0.0567 (11)	0.0536 (12)	−0.0134 (8)	0.0012 (8)	−0.0056 (9)
N1	0.0501 (9)	0.0478 (8)	0.0359 (8)	−0.0031 (7)	0.0117 (7)	0.0011 (6)

### Geometric parameters (Å, °)

**Table:**

P1—O1	1.4961 (11)	C8—C13	1.398 (2)
P1—C14	1.8091 (17)	C9—C10	1.383 (2)
P1—C8	1.8093 (15)	C9—H9A	0.9300
P1—C1	1.8550 (16)	C10—C11	1.367 (3)
O2—C1	1.4140 (18)	C10—H10A	0.9300
O2—H2A	0.8200	C11—C12	1.382 (3)
O3—N1	1.2180 (19)	C11—H11A	0.9300
O4—N1	1.219 (2)	C12—C13	1.388 (2)
C1—C2	1.516 (2)	C12—H12A	0.9300
C1—H1A	0.9800	C13—H13A	0.9300
C2—C3	1.387 (2)	C14—C19	1.384 (2)
C2—C7	1.402 (2)	C14—C15	1.390 (2)
C3—C4	1.391 (3)	C15—C16	1.377 (3)
C3—H3A	0.9300	C15—H15A	0.9300
C4—C5	1.365 (3)	C16—C17	1.373 (3)
C4—H4A	0.9300	C16—H16A	0.9300
C5—C6	1.374 (3)	C17—C18	1.370 (3)
C5—H5A	0.9300	C17—H17A	0.9300
C6—C7	1.384 (2)	C18—C19	1.378 (3)
C6—H6A	0.9300	C18—H18A	0.9300
C7—N1	1.470 (2)	C19—H19A	0.9300
C8—C9	1.388 (2)		
			
O1—P1—C14	112.08 (7)	C10—C9—H9A	119.5
O1—P1—C8	109.96 (7)	C8—C9—H9A	119.5
C14—P1—C8	109.99 (7)	C11—C10—C9	120.14 (18)
O1—P1—C1	111.99 (7)	C11—C10—H10A	119.9
C14—P1—C1	104.15 (7)	C9—C10—H10A	119.9
C8—P1—C1	108.49 (7)	C10—C11—C12	119.96 (17)
C1—O2—H2A	109.5	C10—C11—H11A	120.0
O2—C1—C2	110.46 (13)	C12—C11—H11A	120.0
O2—C1—P1	107.20 (11)	C11—C12—C13	120.53 (17)
C2—C1—P1	107.24 (10)	C11—C12—H12A	119.7
O2—C1—H1A	110.6	C13—C12—H12A	119.7
C2—C1—H1A	110.6	C12—C13—C8	119.72 (16)
P1—C1—H1A	110.6	C12—C13—H13A	120.1
C3—C2—C7	116.01 (15)	C8—C13—H13A	120.1
C3—C2—C1	118.65 (15)	C19—C14—C15	118.46 (16)
C7—C2—C1	124.84 (14)	C19—C14—P1	117.98 (14)
C2—C3—C4	121.67 (18)	C15—C14—P1	123.54 (12)
C2—C3—H3A	119.2	C16—C15—C14	120.60 (17)
C4—C3—H3A	119.2	C16—C15—H15A	119.7
C5—C4—C3	120.57 (18)	C14—C15—H15A	119.7
C5—C4—H4A	119.7	C17—C16—C15	120.1 (2)
C3—C4—H4A	119.7	C17—C16—H16A	120.0
C4—C5—C6	119.74 (18)	C15—C16—H16A	120.0
C4—C5—H5A	120.1	C18—C17—C16	120.00 (19)
C6—C5—H5A	120.1	C18—C17—H17A	120.0
C5—C6—C7	119.51 (18)	C16—C17—H17A	120.0
C5—C6—H6A	120.2	C17—C18—C19	120.23 (19)
C7—C6—H6A	120.2	C17—C18—H18A	119.9
C6—C7—C2	122.46 (16)	C19—C18—H18A	119.9
C6—C7—N1	115.23 (15)	C18—C19—C14	120.63 (19)
C2—C7—N1	122.30 (14)	C18—C19—H19A	119.7
C9—C8—C13	118.62 (15)	C14—C19—H19A	119.7
C9—C8—P1	115.32 (12)	O3—N1—O4	122.76 (16)
C13—C8—P1	126.04 (13)	O3—N1—C7	118.48 (14)
C10—C9—C8	121.00 (16)	O4—N1—C7	118.75 (16)
			
O1—P1—C1—O2	167.18 (10)	C13—C8—C9—C10	0.6 (3)
C14—P1—C1—O2	45.86 (12)	P1—C8—C9—C10	−177.73 (17)
C8—P1—C1—O2	−71.28 (12)	C8—C9—C10—C11	1.1 (3)
O1—P1—C1—C2	48.55 (13)	C9—C10—C11—C12	−1.4 (3)
C14—P1—C1—C2	−72.77 (12)	C10—C11—C12—C13	0.0 (3)
C8—P1—C1—C2	170.09 (11)	C11—C12—C13—C8	1.7 (3)
O2—C1—C2—C3	−14.0 (2)	C9—C8—C13—C12	−1.9 (3)
P1—C1—C2—C3	102.53 (15)	P1—C8—C13—C12	176.16 (14)
O2—C1—C2—C7	174.47 (14)	O1—P1—C14—C19	−21.10 (16)
P1—C1—C2—C7	−69.03 (19)	C8—P1—C14—C19	−143.75 (14)
C7—C2—C3—C4	−0.7 (3)	C1—P1—C14—C19	100.16 (15)
C1—C2—C3—C4	−172.97 (17)	O1—P1—C14—C15	160.53 (15)
C2—C3—C4—C5	1.8 (3)	C8—P1—C14—C15	37.87 (17)
C3—C4—C5—C6	−0.9 (3)	C1—P1—C14—C15	−78.22 (16)
C4—C5—C6—C7	−1.0 (3)	C19—C14—C15—C16	0.2 (3)
C5—C6—C7—C2	2.2 (3)	P1—C14—C15—C16	178.53 (16)
C5—C6—C7—N1	−176.57 (18)	C14—C15—C16—C17	−0.7 (3)
C3—C2—C7—C6	−1.3 (3)	C15—C16—C17—C18	0.5 (4)
C1—C2—C7—C6	170.47 (17)	C16—C17—C18—C19	0.1 (4)
C3—C2—C7—N1	177.34 (16)	C17—C18—C19—C14	−0.7 (3)
C1—C2—C7—N1	−10.9 (3)	C15—C14—C19—C18	0.5 (3)
O1—P1—C8—C9	−1.93 (16)	P1—C14—C19—C18	−177.97 (17)
C14—P1—C8—C9	121.97 (14)	C6—C7—N1—O3	150.69 (18)
C1—P1—C8—C9	−124.70 (14)	C2—C7—N1—O3	−28.0 (2)
O1—P1—C8—C13	179.93 (14)	C6—C7—N1—O4	−28.1 (2)
C14—P1—C8—C13	−56.18 (17)	C2—C7—N1—O4	153.19 (17)
C1—P1—C8—C13	57.15 (17)		

### Hydrogen-bond geometry (Å, °)

**Table:**

*D*—H···*A*	*D*—H	H···*A*	*D*···*A*	*D*—H···*A*
O2—H2A···O1^i^	0.82	1.86	2.6483 (16)	161.
C4—H4A···O3^ii^	0.93	2.52	3.207 (2)	131.
C19—H19A···O2^iii^	0.93	2.50	3.404 (2)	164.

Symmetry codes: (i) *x*+1, *y*, *z*; (ii) −*x*+1, *y*−1/2, −*z*+1/2; (iii) *x*−1, *y*, *z*.
